# Supplementary material for: Splice-Junction-Based Mapping of Alternative Isoforms in the Human Proteome
Source: Cell Rep. Author manuscript; Available in PMC 2020 Jan 15. (PMC6961840; doi:10.1016/j.celrep.2019.11.026)
Supplement: 2 [file NIHMS1546469-supplement-2.zip › DF1/PXD010154/Heart/Heart_67_CYB5R1_QDILFSQISLSILRDAAHHR.pdf]

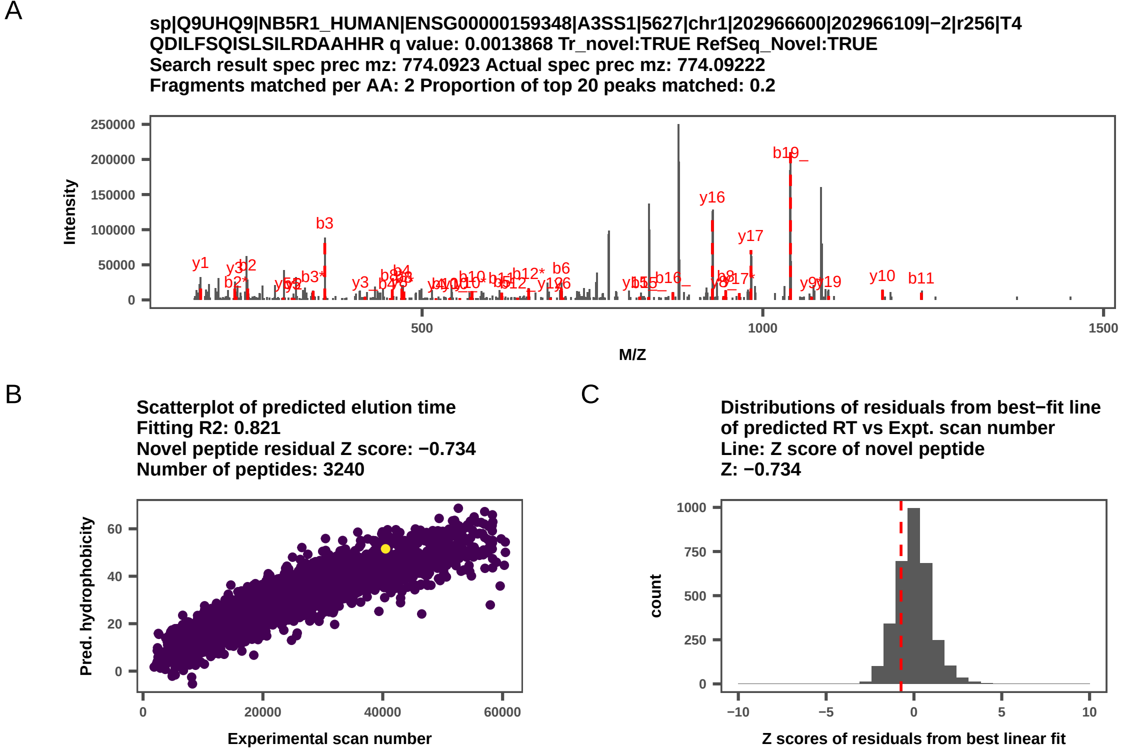
A
sp|Q9UHQ9|NB5R1_HUMAN|ENSG00000159348|A3SS1|5627|chr1|202966600|202966109|−2|r256|T4
QDILFSQISLSILRDAAHHR q value: 0.0013868 Tr_novel:TRUE RefSeq_Novel:TRUE
Search result spec prec mz: 774.0923 Actual spec prec mz: 774.09222
Fragments matched per AA: 2 Proportion of top 20 peaks matched: 0.2
Intensity
250000
200000
150000
100000
50000
0
M/Z
500
1000
1500
y1
b3
y16
y17
b19_
b6
y10
b11
B
Scatterplot of predicted elution time
Fitting R2: 0.821
Novel peptide residual Z score: −0.734
Number of peptides: 3240
Pred. hydrophobicity
60
40
20
0
Experimental scan number
0
20000
40000
60000
C
Distributions of residuals from best−fit line
of predicted RT vs Expt. scan number
Line: Z score of novel peptide
Z: −0.734
count
1000
750
500
250
0
Z scores of residuals from best linear fit
−10
−5
0
5
10
